# Characterizing the Transport and Surface Affinity of Extracellular Vesicles Isolated from Yeast and Bacteria in Well-Characterized Porous Media

**DOI:** 10.1021/acs.est.3c03700

**Published:** 2023-08-22

**Authors:** Nicholas M. K. Rogers, Ethan Hicks, Christopher Kan, Ethan Martin, Lijia Gao, Clariss Limso, Christine Ogilvie Hendren, Meta Kuehn, Mark R. Wiesner

**Affiliations:** † Department of Mechanical Engineering, Porter School of Earth and Environmental Studies, Tel Aviv University, Tel Aviv 69978, Israel; ‡ Center for the Environmental Implications of Nanotechnology, Department of Civil & Environmental Engineering, Duke University, Durham, North Carolina 27708, United States; § Department of Civil & Environmental Engineering, Duke University, Durham, North Carolina 27708, United States; ∥ Department of Biochemistry, Duke University Medical Center, Durham, North Carolina 27710, United States; ⊥ Department of Geological and Environmental Sciences, Research Institute for Environment, Energy and Economics, Appalachian State University, Boone, North Carolina 28608, United States

**Keywords:** extracellular membrane vesicle, colloid, fate, transport model, surface chemistry

## Abstract

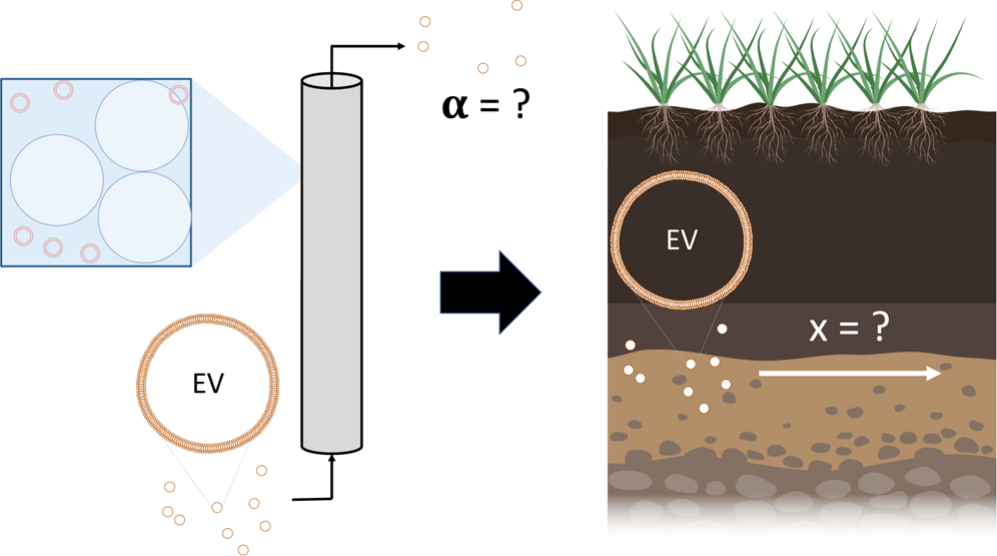

Extracellular vesicles (EVs) are membrane-bounded, nanosized particles, produced and secreted by all biological cell types. EVs are ubiquitous in the environment, operating in various roles including intercellular communication and plant immune modulation. Despite their ubiquity, the role of EV surface chemistry in determining transport has been minimally investigated. Using the zeta (ζ)-potential as a surrogate for surface charge, this work considers the deposition of EVs from the yeast, *Saccharomyces cerevisiae*, and two bacterial species, *Staphylococcus aureus* and *Pseudomonas fluorescens*, in well-characterized porous medium under various background conditions shown to influence the transport of other environmental colloidal particles: ionic strength and humic acid concentration. The affinity of *S. cerevisiae* EVs for the porous medium (glass beads) appeared to be sensitive to changes in ionic strength, as predicted by colloid stability (Derjaguin, Landau, Verwey, and Overbeek or DLVO) theory, and humic acid concentration, while *P. fluorescens* EVs deviated from DLVO predictions, suggesting that mechanisms other than charge stabilization may control the deposition of *P. fluorescens*. Calculations of attachment efficiency from these deposition studies were used to estimate EV transport using a clean-bed filtration model. Based on these calculations, EVs could be transported through such homogeneous porous media up to 15 m.

## Abstract 2

By treating microbe-derived extracellular vesicles as colloidal particles and using columns of saturated porous media, colloid filtration theory is used to estimate minimum distances across which microorganisms might communicate in the environment.

## 1 Introduction

Extracellular vesicles (EVs) are nanoscale (typically 20–500 nm) membrane-bounded particles consisting of diverse proteins, lipids, nucleic acids, and metabolites, produced and released into the environment by all cell types.^1−3^ As cells are ubiquitous in almost every environmental compartment, so are their EVs. From eliciting plant immune responses^4^ to scavenging nutrients for their parent cells,^5,6^ EVs can mediate intercellular and interorganismal communication in the environment, properties integral to microbial systems and the ecosystems in which they exist. Yet, despite their importance, EV environmental transport has been only minimally explored and for a very limited series of organisms. Bos et al. tracked the diffusion of *Escherichia coli*-derived EVs using fluorescence microscopy, demonstrating that these EVs tended to remain close to the parent cells when the parent cells were not under stresses such as extreme environmental conditions.^7^ The examination of mesenchymal stem cell-derived EV transport across a hydrogel barrier by Lenzini et al. showed that these EVs were released from hydrogels at a higher rate than liposomes of a similar size and lipid content.^8^ Only a few studies have examined long-range EV transport from a colloidal perspective, especially regarding EV deposition. To address this shortcoming, this work seeks to examine EV transport patterns experimentally and quantifiably for three microbial EVs using methods and theory drawn from traditional colloidal studies.

The recent use of classical colloidal analysis techniques to investigate EV transport has begun to yield insight into the physical–chemical factors contributing to EV dispersion and stability in the environment. For example, reports characterizing the ζ-potential of human choriocarcinoma cell EVs noted that the EV ζ-potential was sensitive to changes in pH and the presence of multivalent ions but largely unaffected by increasing concentrations of monovalent ions.^9^ Additionally, we previously evaluated the ζ-potential of EVs from three different microbial organisms in varying environmental conditions, demonstrating that in most cases, the ζ-potential of EVs was largely unaffected by changes in ionic strength but was sensitive to pH and humic acid (HA) concentrations.^10^ It should be noted that these characteristics (e.g., their ζ-potentials being affected by pH or the presence of ions or humic acid in certain concentration ranges) are common for colloids, which supports the treatment of EVs as colloids. Significant differences were also found between the ζ-potentials of the bacterial EVs and those of their respective parent cells. From a study of *E. coli* EVs, Gnopo et al. determined that acidic pHs and high ionic strength increased EV aggregation.^11^ While these studies provide an initial glimpse into the conditions that likely impact interactions between EVs and surfaces in the environment, predicting their transport in the environment will be challenging unless the surface properties of EVs are more thoroughly interrogated.

To characterize the transport of colloidal particles, researchers have refined methods to quantify particle attachment to surfaces. One such method utilizes column experiments to determine a value for the attachment efficiency, typically referred to as α.^12−14^ The parameter α may take on values between 0 and 1, describing the probability of attachment between two surfaces upon contact and typically interpreted as being a function of particle surface properties and the local environmental conditions. The attachment of particles to surfaces described by α differs from the phenomena of adsorption and partitioning, which assume thermodynamic equilibrium between particle adsorption and desorption. The complexity of environmental and physiological systems currently require the experimental determination of α rather than being calculated from colloid stability theory, such as Derjaguin, Landau, Verwey, and Overbeek (DLVO) theory.^15,16^ Experimental estimates of α can be obtained from column studies (deposition studies) in which a suspension of the investigated particles is passed through a column of porous medium and the influent and effluent particle concentrations across the porous medium are combined with theoretical estimates of particle collision rates (or normalized by a case where α = 1), to obtain an estimate of the attachment efficiency, α.^17^ This approach has been used primarily for a wide variety of colloids^18−22^ including some biotic particles.^23−28^ The parameter α has been evaluated in both lab settings and in field work and has been shown to be an excellent predictor of environmental fate when used in concert with population balances.^12−14,29,30^ By applying a similar approach to EVs, our goal here is to obtain estimates of a “sphere of influence” that a cell may exert in the distribution of EVs in an environmental setting. For example, the distance across which EVs from a microbe might reach plant root systems in agriculture or the maximum transport distance that EVs carrying nutrients could be delivered in a groundwater system could be predicted by means of α experiments and modeling. Such estimates however must be treated as relative indicators due to the influence that heterogeneity may have in an actual environmental setting.

Relying on the success of column studies to characterize particle deposition, the goal of this work is to characterize α for EVs under various conditions of solution chemistry, specifically changes in ionic strength and the presence of a naturally occurring organic matter, HA. These environmental factors were specifically chosen to allow this work to build on previously published ζ-potential data for EVs under similar environmental conditions.^10^ These evaluations allow for a comparison between observations of the ζ-potential and trends in attachment efficiency, α, and consequently inform a deeper understanding of the role electrostatics play in EV deposition. The values of α obtained from these column studies were used to determine the collector efficiency of the EV and a glass bead collector^17,31^ in a clean-bed filter model.^32,33^ Finally, the collector efficiency was used to predict EV transport through a hypothetical column of saturated porous media where the relative influence of HA concentration and ionic strength on EV transport can be compared. Collectively, α values and the associated model will allow for initial predictions for transport distances of EVs in the environment and highlight conditions that might facilitate or prevent further transport.

## 2 Materials and Methods

### 2.1 Organisms, Growth Conditions, and Reagents

The three model organisms used in this study were grown in liquid cultures to a stationary phase with shaking, as previously described in Rogers et al.^10^ Briefly, the *Saccharomyces cerevisiae* strain YEF473 was grown at 30 °C overnight in liquid yeast nitrogen base (YNB) media (Sigma-Aldrich; St. Louis, MO) supplemented with 0.79 g/L complete synthetic media (Sunrise Science Products; San Diego, CA) and 20 g/L dextrose. *Pseudomonas fluorescens* Migula ATCC 13525 was grown in liquid King’s broth (KB) media (2% proteose peptone, 8.6 mM K_2_HPO_4_, 1.4% glycerol, 6 mM MgSO_4_) at 30 °C overnight. *Staphylococcus aureus* Newman was grown in nutrient broth (NB) media (0.5% proteose peptone, 0.3% beef extract) at 37 °C overnight. Stock solutions of 1× PBS (Sigma-Aldrich; P3813) were prepared and diluted to an ionic strength of 1, 10, 25, or 50 mM with nanopure water. Unless otherwise specified, chemicals and reagents were purchased from Sigma-Aldrich, St Louis, MO.

### 2.2 Vesicle Isolation and Sample Preparation

EVs were isolated with modifications to the protocol in McMillan et al. and Rodriguez and Kuehn.^4,34^ To remove cells, cultures were centrifuged (Eppendorf 5804R, Rotor: FA-45-6-30; 10,000*g*, 30 min, 4 °C). Supernatants were then filtered with a 0.45 μm poly(ether sulfone) (PES) filter to ensure that all cells were removed. EVs were then concentrated using tangential flow filtration with a 100 kDa cutoff filter (Pall Corporation, Port Washington, NY) and then filtered again using a 0.45 μm PES syringe filter (Pall Corporation, Port Washington, NY, P: 60206). Finally, EVs were pelleted via centrifugation (Beckman Optima TLX ultracentrifuge, Rotor: TLA-100.3; 90,935*g*, 1 hr, 4 °C) and resuspended in 50 mM PBS. EV samples were stored at 4 °C and analyzed shortly thereafter.

### 2.3 EV Quantification by the Bradford Assay and Sample Preparation

Ten microliters of each of the final EV stocks was added to 300 μL of Bradford reagent (VWR, Radnor, Pennsylvania) in duplicate, and the absorbance was read at 595 nm. To determine the protein concentrations of the EV stocks, these readings were compared with the standard linear curve generated using a series of bovine serum albumin concentrations. To prepare samples for particle imaging and analysis, EV stocks were diluted with deionized water to a protein concentration of 2 μg/mL and supplemented with the appropriate amount of 1× PBS and/or humic acid (HA) solution to adjust to the desired levels. A stock of 50 mg/L Pahokee Peat standard humic acid (International Humic Substance Society [IHSS], cat. #1S103H) was used as the HA solution, which is a standard agricultural soil humic acid, following a similar protocol to Rogers et al.^10^ The initial pH value was between 6 and 8, depending on initial solution composition and was adjusted to pH 7.0 ± 0.2 using 0.1 M HCl or NaOH with mixing. This addition of acid or base was determined to be less than 10% of the total ionic strength and follows a similar protocol to Rogers et al.^10^

### 2.4 Size and ζ-Potential Evaluation

Size determinations were performed using a Malvern ZetaSizer ZS (Malvern, U.K.), as in Rogers et al., and a transmission electron microscope (TEM). The light intensity distribution from the dynamic light scattering (DLS) data comes from nine measurements (triplicate measurement of three biological replicates). EVs were prepared for TEM imaging by deposition on formvar/carbon grids (400 mesh, copper). 15 μL of sample was pipetted onto the grid, left for 20 min, and the remaining liquid was gently wicked away. Then, 5 μL of vanadium negative stain (Abcam, ab172780, Cambridge, U.K.) was placed on the grid and left for 10 min, and the remaining stain was wicked off. The grid was rinsed with 15 μL of nanopure water and air-dried for 2 h. TEM imaging was performed using an JEOL TEM 2100 Plus microscope (JEOL; Japan) with a controlled exposure time between 7 and 8 s. Images were then processed for size using the NIH’s Image J software.

Electrophoretic mobility measurements (which were then converted to the ζ-potential using the Henry equation^35^) were conducted also using the same Malvern ZetaSizer as was used for the size measurements. The sample settings for refractive index (1.330) and absorption (0.060) for liposomes were used for ζ-potential calculations. The initial pH value was between 6 and 8, depending on the initial solution composition, and was adjusted to pH 7.0 ± 0.2 using 0.1 M HCl or NaOH with mixing. Standard error is reported to compare the mean values of the ζ-potential between the different organisms and their conditions.

### 2.5 Glass Bead Preparation

Spherical glass beads were used as porous medium in this work. To prepare glass beads, methods from Pelley and Tufenkji were followed.^36^ In this method, 1 L batches of 360 μm glass beads were soaked in 12N HCl for 24 h. After acid washing, the beads were rinsed thoroughly with deionized water until the pH of the decanted liquid from the washing was at least 5.6 to ensure that all acid was removed from system. Beads were transferred to crucibles and baked for an hour at 120 °C to evaporate all water. Beads were then baked at 600 °C for 5 h to remove all organic content from the surface of the packing materials. The cleaned materials were stored in a clean, airtight container until use.

### 2.6 Column Description and Operation

A schematic of the column apparatus is provided in Figure S1. Briefly, the column testing setup was arranged as follows: 13 g ± 0.1 g of dry glass beads were packed into a glass column (10 mm × 150 mm, Diba Omnifit EZ Chromatography Column, Cole Parmer). Deionized water was pumped into the column from the bottom by means of a syringe pump until the whole column was filled with water. The column effluent was fed into an in-line ultraviolet–visible (UV–vis) spectrophotometer (Evolution 201 UV–visible Spectrophotometer, CAT# 912A0883, Thermo Fischer Scientific), and the spectrophotometer effluent was discarded. Roughly 10 pore volumes of the background electrolyte (i.e., PBS or PBS with HA) was passed through the column to equilibrate the packing material with the electrolyte, following established protocols.^14,37^ The spectrophotometer was blanked to the background electrolyte. Then, the EV sample was passed directly into the spectrophotometer, bypassing the column by means of T-connectors, to obtain a maximum absorbance at 280 nm for the sample (the initial EV concentration, *C*_0_). The tubing was then flushed with more background electrolytes to remove all of the EV sample from the system. The EV sample was then passed directly through the column at a flow rate of 0.8 mL/min, and the absorbance at 280 nm was recorded at approximately every 10 s for at least four bed volumes (i.e., until the volume passed through the packed bed was equal to 4 times the volume of the packed bed). Experiments where EV type, ionic strength, and HA concentration were varied were conducted at a flow rate of 0.8 mL/min, which corresponds to a Darcy velocity of 0.017 cm/s.

A tracer study using potassium nitrate was performed to evaluate the integrity of the column and the residence times of associated connections (Figure S2). Tracer tests verified the residence time of the column and indicated that the system plateaued (i.e., the signal demonstrated that the packed bed could no longer “collect” more particles) after approximately 2.25 bed volumes, indicating the clean-bed volume where the peak *C*/*C*_0_ values for the model were to be taken. The details of how the tracer study was performed are included in the Supporting Information.

### 2.7 Calculation of Attachment Efficiency (α)

Classic filtration theory was used as the framework for interpreting particle removal by the saturated porous medium in terms of the attachment efficiency. Particle concentration as a function of the length of the porous medium is derived from a mass balance across the medium, with deposition occurring as a first-order decay with respect to the distance^38^
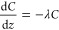
1where *C* is the concentration of particles (EVs), *z* is the distance through the porous medium, and λ is the first-order deposition rate constant or filtration coefficient. This equation can be integrated by using a relationship for λ, which is expressed as a function of the single collector efficiency, η_0_, the attachment efficiency, the diameter of a single collector (e.g., a single glass bead), and the porosity of the porous medium.^23^ Substituting for the filtration coefficient and rearranging the attachment efficiency can be expressed as a function of the experimentally observed concentrations of particles in the porous medium effluent, *C* and influent *C*_0_
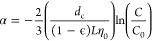
2where *d*_c_ is the collector (glass bead) diameter, ϵ is the porosity, and *L* is the length of the bed. η_0_ can be calculated from the various parameters for the system based on the equations provided in Tufenkji and Elimelech;^17^ tabulated parameters used in these calculations are listed in Table S1. The value of α was determined using eq 2, with the value of *C*/*C*_0_ taken from the steady-state phase of the column data. Experiments for α were conducted in duplicate, and standard error is reported to compare between different experiments.

### 2.8 Model Validation

To predict the transport distance of EVs, eq 2 was rearranged to solve for the column length
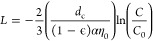
3

Maintaining α as a constant, eq 3 was solved for a variety of lengths *L*, across which an EV may be transported. Eq 3 was solved analytically in MATLAB, R2019a 64-bit academic use license, on a 2018 MacBook Pro laptop. The MATLAB code and a full table of collector efficiency parameter values can be found in the Supporting Information.

To validate the clean-bed filtration model used as the basis for calculating attachment efficiency for these vesicles, additional column experiments were performed at different flow rates, specifically at 0.2, 0.4, and 0.8 mL/min. These comparisons were only performed for *S. cerevisiae* EVs in 1 mM PBS and without HA, which correspond to the same parameters inputted into the model.

### 2.9 Statistical Analysis

Statistical analysis was conducted for both the ζ-potential and *C*/*C*_0_ results as a function of media conditions and the organism from which the EVs were produced. To test for significance, one-way analysis of variance (ANOVA) and subsequent pairwise testing with Tukey’s honestly significant difference (HSD) test was performed in MATLAB for each organism’s EVs and for both changes in ionic strength and HA concentration separately. The same statistical test was performed for *C*/*C*_0_ results for different Darcy velocities, using one-way ANOVA and Tukey’s HSD tests. The tabulated results of the ANOVA and Tukey’s HSD tests are included in the Supporting Information.

## 3 Results and Discussion

### 3.1 Characterization of EVs

EVs were collected from the pelleted fraction of culture supernatants from *S. cerevisiae*, *S. aureus*, and *P. fluorescens*. The size of the EVs was determined by DLS and TEM to both validate the presence of EVs and to identify any difference in size that may influence transport (Table 1). The light intensity distribution from the DLS data is presented in Figure S5. TEM images confirmed the presence of EVs (Figure S6), showing similar features to previous reports.^39,40^ The ranges of ionic strength and humic acid (HA) were chosen to reflect conditions commonly found in the environment and to compare with previous results.^10^

**Table 1:** EV Size Ranges and ζ-Potential Values

	TEM		DLS	ζ-potential^c^					
				ionic strength			humic acid concentration		
organism	average diameter^a^ (nm)	total counts	Z-average diameter^a^^,^^b^ (nm)	1 mM 0 mg/L HA	10 mM 0 mg/L HA	25 mM 0 mg/L HA	10 mM 0 mg/L HA	10 mM 1 mg/L HA	10 mM 10 mg/L HA
*P. fluorescens*	81.2 ± 31.8	42	171.7 ± 32.6	–12.9 ± 4.7A	–4.6 ± 0.5B	–6.8 ± 1.7B	–4.6 ± 0.5A	–6.9 ± 2.1B	–8.2 ± 2.6B
*S. aureus*	75.1 ± 37.2	50	193.0 ± 70.8	–22.5 ± 5.2A	–15.8 ± 4.9A	–14.8 ± 4.0A	–15.8 ± 4.9A	–19.0 ± 3.8B	–26.4 ± 5.8B
*S. cerevisiae*	133.1 ± 67.4	51	212.3 ± 16.7	–11.0 ± 0.4A	–4.3 ± 0.9B	–1.5 ± 0.5C	–4.3 ± 0.9A	–5.6 ± 1.2B	–8.4 ± 3.0B

a Error is standard deviation for both measurement methods.

b The Z-average diameter is the intensity-weighted, harmonic mean size. DLS error is based on three biological replicates (EVs were isolated from three independent cultures for each organism).

c All ζ-potential values are reported at pH = 7.0 ± 0.2. ζ-potential values were compared for EVs derived from the same organism in the indicated ionic strengths and HA concentrations. Distinct letters indicate statistically different values. Statistical differences between different organisms’ EVs were not evaluated.

As shown in Table 1, measurements using TEM of all three types of EVs revealed a lower measured particle diameter compared to the measurements with DLS. This is consistent with previous research, in particular with TEM tending to underestimate EV size while DLS tending to overestimate EV size.^41^ In addition to these size measurements, the TEM images show that there is a coating on the *S. aureus* EVs (Figure S6c). It is likely that this coating is a concentrated extracellular material from the tangential filtration step of the EV preparation methods. While this material may associate with the EVs upon dehydration, this material may be contributing to the size distribution of size measurements within the samples.

### 3.2 Breakthrough Curves and α Determination

**Figure 1:**
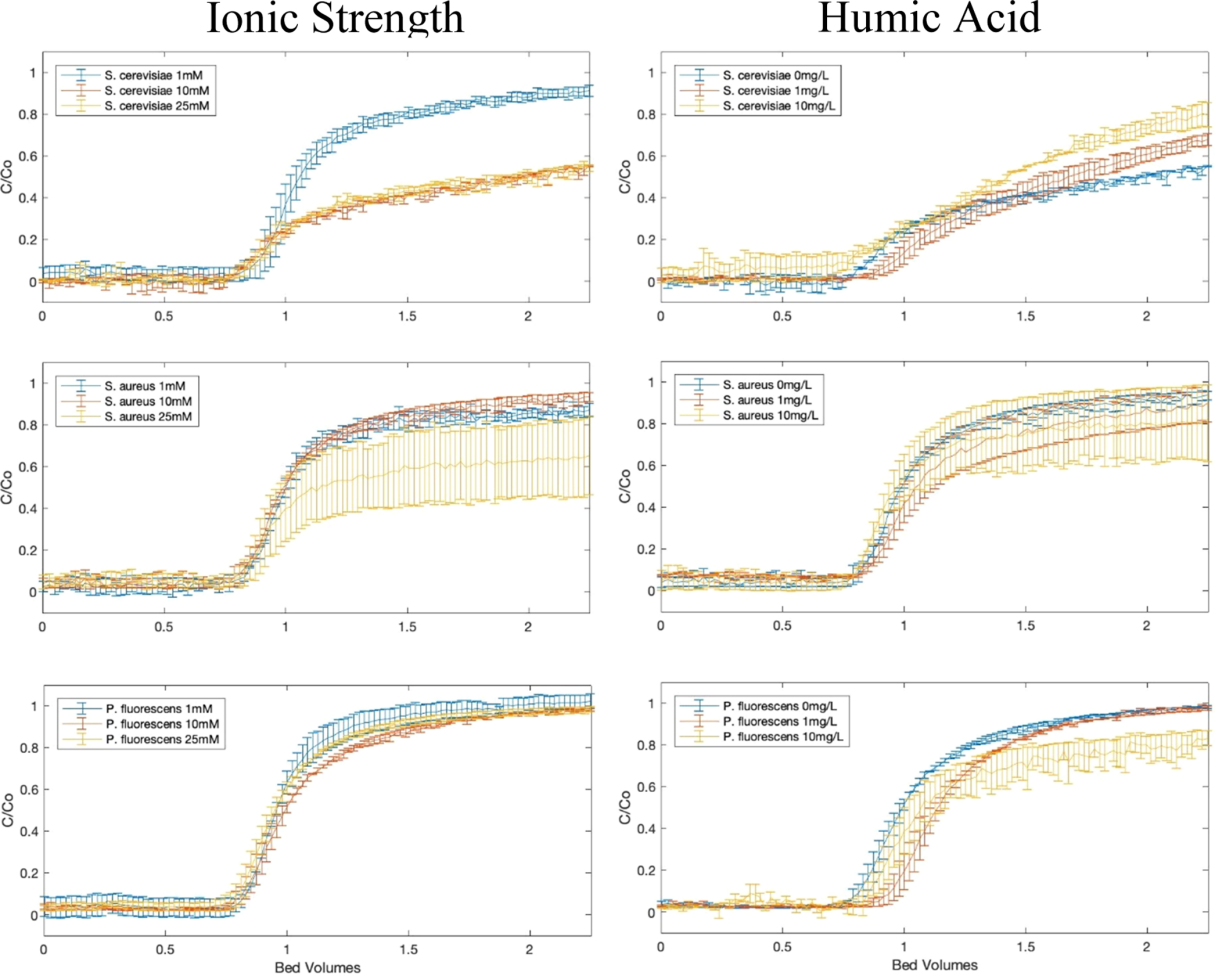
Breakthrough curves for three EVs as a function of PBS ionic strength (left panels) and HA concentration (right panels). Breakthrough curves are measured at pH 7. The error bars are standard error for duplicate measurements. The indicated ionic strength (in mM) is the ionic strength of PBS; the indicated mass concentration (in mg/L) is the concentration of HA. Data graphed on the left column were all with 0 mg/L HA; data graphed on the right were all measured using 10 mM PBS conditions.

To determine the transport capabilities of EVs, column analyses were performed for all three EV types using various ionic strength and HA conditions (Figure 1). The curves shown here are typically referred to as “breakthrough curves”, where the analyte (EVs, in this case) becomes detectible in the effluent. Here, EVs are first detected around 1 bed volume (the point of “breakthrough”) and are detected until an equilibrium between deposited particles onto the packed media and the suspended particles is reached. Experimentally, this equilibrium is observed when the normalized concentration reaches a plateau with respect to time. While not all breakthrough curves indicate this equilibrium (i.e., there is no plateau), the equilibrium point is determined by a tracer experiment (Figure S2), and for this study, the equilibrium point was reached at 2.25 bed volumes. The normalized concentrations for all breakthrough curves at this plateau point are shown in Tables 2 and 3.

**Table 2:** Calculated Values of α for Three Populations of EVs in Various Ionic Strengths

organism	ionic strength (mM)	*C*/*C*_0_^a^^,^^b^	α^a^
*S. cerevisiae*	1	0.91 ± 0.028A	0.0050 ± 0.0017
	10	0.55 ± 0.0025B	0.032 ± 0.00024
	25	0.54 ± 0.015B	0.033 ± 0.0015
*S. aureus*	1	0.87 ± 0.031A	0.0074 ± 0.0019
	10	0.94 ± 0.022A	0.0036 ± 0.0013
	25	0.66 ± 0.19A	0.025 ± 0.016
*P. fluoresceins*	1	1.02 ± 0.034A	0.00032 ± 0.00032
	10	0.98 ± 0.0076A	0.00096 ± 0.00042
	25	0.98 ± 0.012A	0.00088 ± 0.00065

a Error is standard error based on duplicate measurements.

b *C*/*C*_0_ values were compared for EVs derived from the same organism. Distinct letters indicate statistically different values. Statistical differences between different organisms’ EVs were not evaluated.

**Table 3:** Calculated Values of α for Three Populations of EVs in Various HA Concentrations

organism	humic acid concentration (mg/L)	*C*/*C*_0_^a^^,^^b^	α^a^
*S. cerevisiae*	0	0.55 ± 0.0025A	0.032 ± 0.00024
	1	0.69 ± 0.022B	0.020 ± 0.0018
	10	0.80 ± 0.059B	0.012 ± 0.0040
*S. aureus*	0	0.94 ± 0.022A	0.0036 ± 0.0013
	1	0.90 ± 0.089A	0.0060 ± 0.0054
	10	0.80 ± 0.18A	0.013 ± 0.013
*P. fluorescens*	0	0.98 ± 0.0076A	0.00096 ± 0.00042
	1	0.98 ± 0.011A	0.0013 ± 0.00063
	10	0.83 ± 0.036B	0.010 ± 0.0024

a Error is standard error based on duplicate measurements.

b *C*/*C*_0_ values were compared for EVs derived from the same organism. Distinct letters indicate statistically different values. Statistical differences between different organisms’ EVs were not evaluated.

According to ANOVA, only the *C*/*C*_0_ values for *S. cerevisiae* EVs were affected by changes in ionic strength. Tukey’s HSD test showed that the plateau for *S. cerevisiae* EVs in 1 mM PBS was significantly different from those in 10 or 25 mM PBS. Similarly, ANOVA shows that ζ-potential values for both *S. cerevisiae* and *P. fluorescens* EVs were affected by changes in ionic strength. Tukey’s HSD shows that the ζ-potential for *P. fluorescens* EVs at 1 mM PBS is significantly different than at 10 or 25 mM PBS, while the ζ-potential at all ionic strengths for *S. cerevisiae* EVs are significantly different. ANOVA also indicated that both *S. cerevisiae* and *P. fluorescens* EVs were affected by changes in HA concentration, regarding C/C_0_ values. Tukey’s HSD test indicated that the plateau for *P. fluorescens* EVs in 10 mg/L HA was significantly different from those in 0 or 1 mg/L HA. For *S. cerevisiae* EVs, Tukey’s HSD only indicated that the *C*/*C*_0_ value for the 0 mg/L solution of HA was different from the 10 mg/L sample. For ζ-potential values, ANOVA indicated that all EV types were affected by HA concentration, and Tukey’s HSD showed that the ζ-potential for all EV types at 0 mg/L HA was significantly different from the ζ-potential with 10 mg/L HA.

Looking first at trends for the influence of ionic strength on EV deposition, expectations based on trends predicted by DLVO are that particle deposition should be favored at a higher ionic strength.^26,36,42−46^ From Figure 1, we noted that none of the EV populations deposited on the collector particles to a high extent at low ionic strength (1 mM). As ionic strength increased, different trends emerged for the different EV populations. For *S. cerevisiae* EVs, as ionic strength increased to 10 or 25 mM, their deposition to collector particles,and thus their attachment efficiency increased significantly, based upon both the standard error and Tukey’s HSD test for *C*/*C*_0_ values. A substantially lower *C*/*C*_0_ value was also observed for *S. aureus* EVs at the highest ionic strength (25 mM), though this difference was not significantly different according to ANOVA. These results were corroborated with the ζ-potential data: as ionic strength increased, the surface potential of *S. cerevisiae* and *S. aureus* EVs became less negative, implying that the electrostatic repulsions between particles and collectors would be reduced to allow them to deposit to a greater extent. This observation is predicted by DLVO theory, and it follows a similar trend described in previous research regarding the effect of ionic strength on particle deposition, both for anthropogenic particles^36,42−44^ and other biocolloids.^26,45,46^ A similar destabilization could be seen in the less negative ζ-potential for *P. fluorescens* EVs at higher ionic strengths, but this effect was not observed for the deposition on collectors, as represented by the similar *C*/*C*_0_ values. This implies that there may be other factors influencing the deposition of *P. fluorescens* EVs, such as steric effects from other extracellular materials, hydrophobic effects, or unique interactions from the presence of lipopolysaccharides (LPS). As a Gram-negative organism, the outer membrane bilayer surface of *P. fluorescens* consists of LPS, and LPS is also a known surface component of its EVs.^47−49^ The external-facing parts of LPS consist of long chains of sugars (O-antigen) that could cause steric stabilization interactions with other EVs. Additionally, O-antigen is known to shield the negative charges of the lipid surface,^11^ making *P. fluorescens* EVs less sensitive to changes in ionic strength. Such shielding would not be the case for Gram-positive or yeast EVs which lack LPS.

Next, we considered the influence of HA in our column studies. HA sorption to particle surfaces is known to impart electrosteric stabilization to particles.^36,42−44,50^ We observed that the presence of the highest tested concentration of HA (10 mg/L) was associated with a greater level of breakthough for *S. cerevisiae* EVs (Figure 1). This trend is consistent with the ζ-potential data. For the highest concentration of HA, the ζ-potential of EVs at neutral pH became more negative (Table 1), which would imply greater electrostatic repulsion between particles. This is reflected in the reduced deposition on the collectors and thus a reduced attachment efficiency too. This result also aligns with existing research that shows the stabilizing effect of HA on colloidal particles.^36,42−44,50^ In addition, previous work shows that lower concentrations (<2 mg/L) of HA have a less pronounced effect on colloidal deposition,^50^ as also reported in this study.

By contrast, deposition of the *P. fluorescens* EVs increased in the presence of HA resulting in a lower *C*/*C*_0_ value, and there was no significant effect on C/C_0_ for *S. aureus* EVs with respect to HA concentrations. We recall that for all three organisms, this higher concentration of HA resulted in a more negative ζ-potential at neutral pH (Table 1); thus, the effects of HA on EV stability cannot be explained entirely based on charge stabilization. That the particle deposition of *P. fluorescens* EVs increases with increasing HA concentration was particularly surprising: according to the ζ-potential data, a 10 mg/L addition of HA caused the ζ-potential to be more negative relative to the *P. fluorescens* EVs without HA, meaning that some factor other than electrostatic forces must be influencing the deposition of EVs. LPS has been shown to play a role as adhesins in biofilm formation.^51^ In addition, LPS has been shown to interact with HA,^52^ which collectively may be the cause of this increased attachment. This departure from classic DLVO theory demonstrates that the transport of EVs must be evaluated with more than surface potential measurements. Unlike the attachment efficiencies of *P. fluorescens* EVs, the attachment efficiencies of *S. aureus* EVs are not significantly different. The lack of LPS on the surface of Gram-positive bacterial EVs could be the reason for this difference between bacterial species.

To make comparisons to other colloidal systems for which attachment efficiencies are known and to parameterize the transport model, α was calculated using eq 2, where the C/C_0_ values used were those recorded at 2.25 bed volumes. Tables 2 and 3 present those calculated values. Other parameter values used in eq 2 to calculate α can be found in Table S1.

The α values for the three types of EVs we evaluated in this study are comparable to those reported for other biocolloids. For instance, for bacteriophage PRD1, values of α were reported from 0.0011 to 0.0053 at pH 7 for an ionic strength from 1 to 20 mM.^53^ These α values are on the same order of magnitude as those for EVs from *S. cerevisiae* and *S. aureus*, but they are still higher than those for *P. fluorescens*, again highlighting the possible role that the distinct properties of EVs derive from different parent species.

Minimal data exist for how the attachment efficiency of biocolloids varies as a function of the organic content in the surrounding media. One study examined the deposition of *Cryptosporidium parvum* oocysts which showed a reduced α from 0.87 to 0.18 when the organic content was present.^54^ Another similar study of *C. parvum* oocysts revealed that with the addition of 5 mg/L natural organic matter, the value of α dropped from 0.84 to 0.22, implying increased stability.^55^ These trends align with our data for *S. cerevisiae* EVs but not for either of the bacteria-derived EVs.

### 3.3 EV Removal as a Function of Darcy Velocity

**Figure 2:**
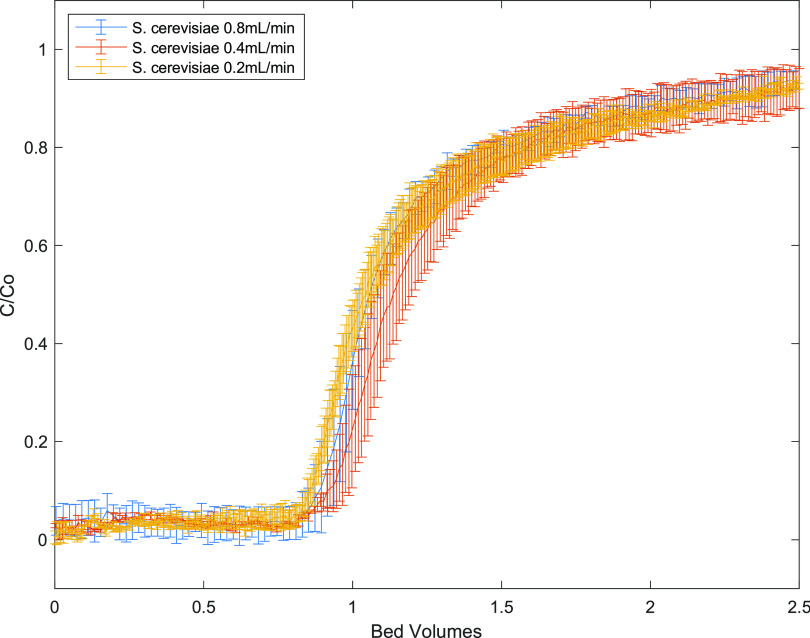
Breakthrough curves for *S. cerevisiae* EVs at three different flow rates in 1 mM PBS at pH 7. The error bars are standard error for duplicate measurements.

The closed form solutions for collector efficiency^17,31^ predict that collector efficiency for a particle of a given size and attachment efficiency should decrease with increasing velocity through the porous medium. Using *S. cerevisiae* EVs, column studies were carried out under a range of superficial flow rates (0.8, 0.4, and 0.2 mL/min) under the assumption that EV attachment efficiency and flow rates are independent of each other. The breakthrough curves for these systems are shown in Figure 2. These flow rates were chosen because this range fits into a typical groundwater flow rate range^56^ and align with previous column experiment flow rates.^37,57^ Given a filter area of 7.85 × 10^–5^ m, the corresponding Darcy velocities of these experiments were 1.7 × 10^–4^, 8.5 × 10^–5^, and 4.2 × 10^–5^ m/s, respectively. According to eq 2, collector efficiency should decrease with increasing velocity through the porous medium.

**Figure 3:**
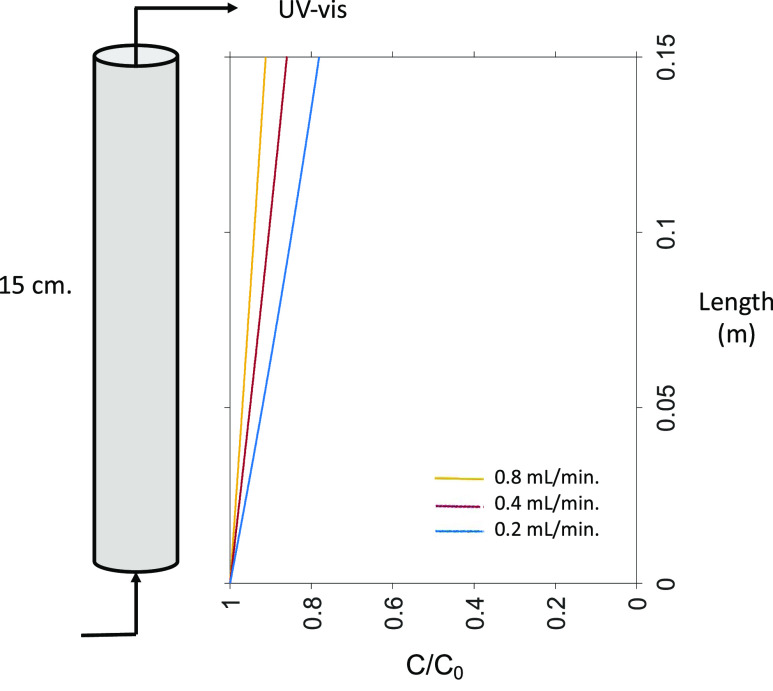
Model-determined normalized concentration profile of *S. cerevisiae* EVs through the column as a function of flow rate in 1 mM PBS at pH 7 with no HA. Model-predicted values of the outlet concentration are listed in Table 4.

Data obtained from experiments were then compared with calculations of anticipated concentrations using eq 2 using an average value of α for *S. cerevisiae* of 0.00496, determined from experiments in the absence of HA and at 1 mM ionic strength and pH 7 (Table 2). All other experimental conditions and model parameters were held constant for the purpose of comparison, changing only the value of the Darcy velocity. Figure 3 shows the results of calculations for the predicted EV concentration profile through the column for each of the flow rates. These calculations indicate that the effluent concentration from the column (normalized to influent concentration) is predicted to drop by approximately 15% as the flow rate decreases from 0.8 to 0.2 mL/min. However, we note that our observed EV effluent concentrations only dropped by approximately 3% (Table 2).

Table 4 compares the experimentally observed removal of EVs with the theoretically determined (model) removal. In general, the theoretical and observed removals were similar, especially in the case of 0.8 and 0.4 mL/min, and the observed trends align with the theoretical expectation that at a lower flow rate, there would be greater removal. However, in the case of the 0.2 mL/min flow rate, a divergence between the observed and expected values is statistically significant. It is noted that the differences in the observed *C*/*C*_0_ values are not statistically significant (Table S8).

**Table 4:** Theoretical and Observed Values of EV Removal^a^

Darcy velocity (m/s) × 10^–5^	superficial flow rate (mL/min)	theoretical *C*/*C*_0_	observed *C*/*C*_0_
17.0	0.8	0.91	0.91 ± 0.02
8.5	0.4	0.86	0.90 ± 0.05
4.2	0.2	0.78	0.89 ± 0.005

a Error is standard error based on duplicate measurements.

Consistent with filtration theory and previous experimental results,^58−60^ the data show that as flow rate decreases, the EV deposition increases. While qualitative trends predicted by theory were observed, a statistically significant difference between predicted and observed differences in particle removal was observed at the lowest Darcy velocity. EV attachment efficiency should not be affected by a changing velocity but the collector efficiency will be. Furthermore, EV size and density are the only variable parameters unique to determining the collector efficiency; hence, estimates for these parameters may need to be better characterized for future iterations of this transport model.

The column experiments and corresponding model presented above may be used as the basis for making rough estimates of the spatial zone of influence that a given source of EVs might exert. This zone of influence would be relevant in predicting, for instance, the distance that a pathogenic organism could affect a target organism, or the distance over which genetic material from one organism might be transported in the environment. Such zones of influence might be considered in reverse-engineering EVs to create delivery vehicles for agricultural supplements, using, for example, EVs as nutrient “taxis” to root systems.

## 4 Estimations of EV Transport in Porous Media

**Figure 4:**
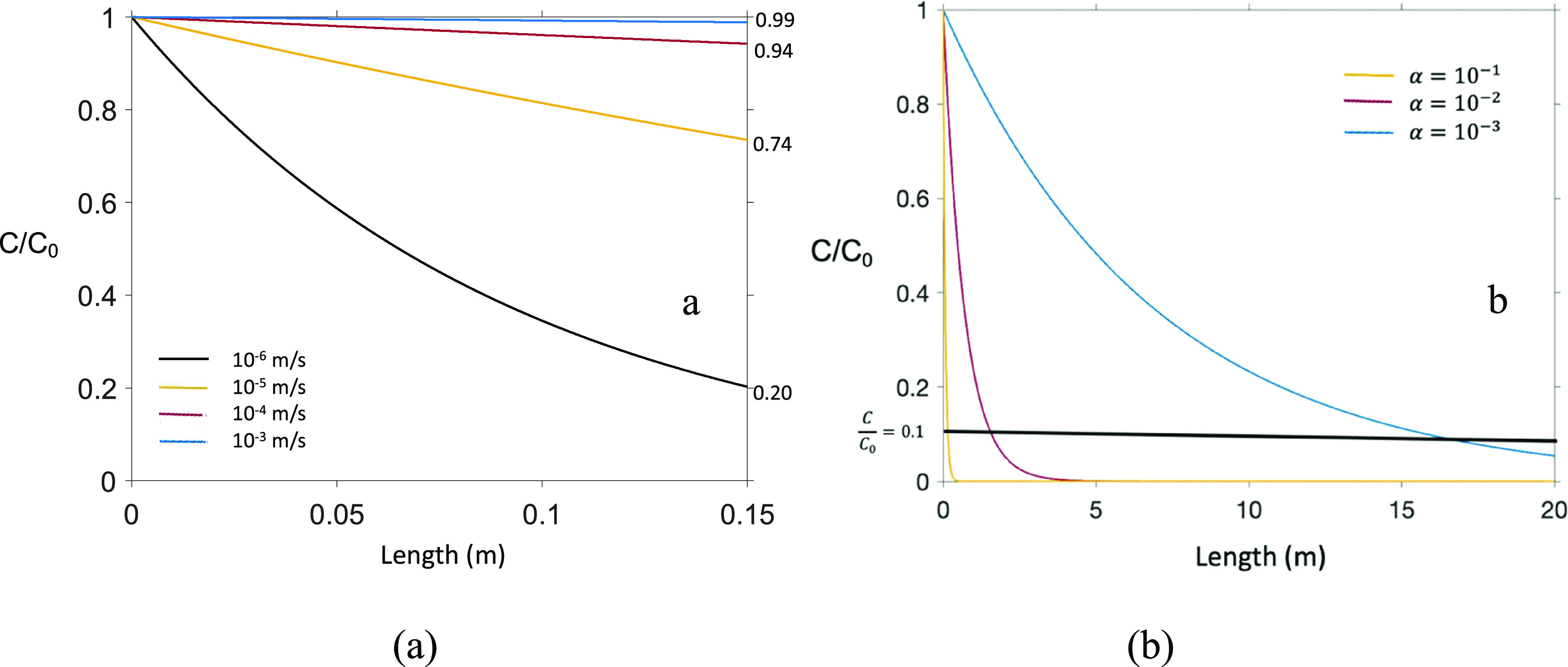
Model prediction for EV transport through saturated porous media. (a) Projected concentration profile at a distance of 0.15 m as a function Darcy velocity. (b) Projected concentration profile for a Darcy velocity of 10^–4^ m/s, noting when *C*/*C*_0_ = 0.1, as a function of α.

By performing sensitivity analyses with this model, initial predictions for long-range transport of EVs can be determined and the model’s limitations can be identified. Figure 4 shows the model output where the fraction of EVs removed are shown for various Darcy velocities and values of α as a function of media depth (i.e., depth of packed, porous material). Figure 4a shows that for a bed depth equal to that of the column, less than 2% of the EVs are removed at a Darcy velocity of 10^–3^ m/s, while at two orders of magnitude greater velocity, there is greater than 25% removal. To contextualize this observation, groundwater Darcy velocities range from 10^–3^ to 10^–6^ m/s.^56^ In addition, for α = 0.1, the model shows that the EV concentration is diminished to 10% of the initial concentration after moving through 0.15 m of saturated porous media, while for an α = 0.001, EV concentration does not drop to 10% until passing through almost 17 m of saturated porous media.

**Figure 5:**
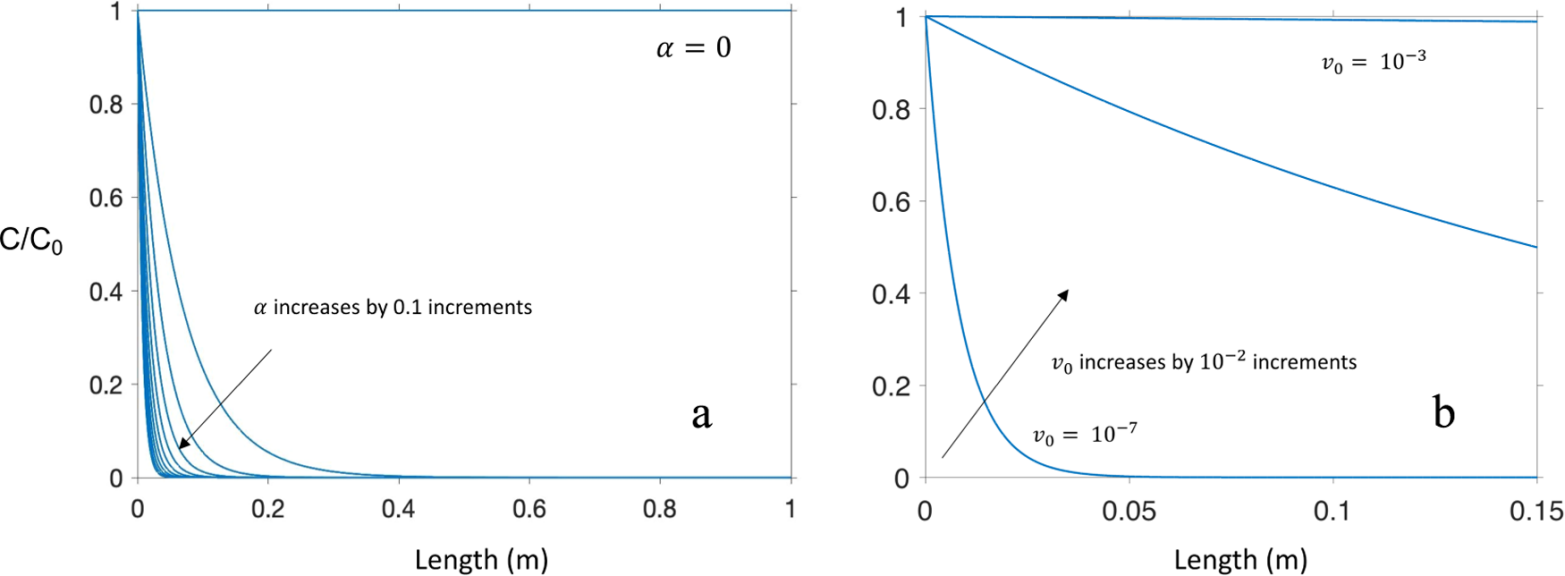
(a) Model sensitivity to changes in α using a Darcy velocity of 5.4 × 10^–5^ m/s. (b) Model sensitivity to changes in flow rate with an α = 0.00496.

Given the interest in some research sectors to think more specifically about engineering EVs to act as shuttles of nutrients^66^ or even specific/desired genetic material, Figure 5a provides a sensitivity analysis for how the deposition of EVs might change in response to alterations to attachment efficiency, a parameter that engineers may be able change through modifications to EV surface properties. Figure 5b shows a sensitivity analysis for changes in model responses to changes in flow rate. While similar calculations for theoretical transport could be applied to other environmentally relevant nanomaterials, to our knowledge, this report contains the first application to EVs. Note that other model parameters were left unchanged in these analyses.

These sensitivity analyses reveal that under both conditional changes, there is a great potential to fine-tune EV transport through columns of saturated porous media. As seen in Figure 5a, even for minuscule increases in attachment efficiency, EV-collector deposition could be discouraged and EV transport increased. The same could be said of flow rate, where the natural changes to rainwater flow, for example, could dramatically alter the expected transport of EVs.

The transport of EVs depends on their environmental conditions such as solution chemistry. DLVO theory can help us to predict this transport, but some of the trends observed in this work deviated from theoretical expectations. The transport of EVs from the Gram-negative bacteria *P. fluorescens* seemed to consistently differ from expectations based on surface potential measurements, with many of the unique behaviors possibly stemming from the presence of LPS on the surface of their EVs. Continuing to expand the study of organisms’ EVs beyond the three species evaluated here will help to make broader claims about EV transport. In addition, the effects of upstream isolation methods on downstream surface property analysis are necessary for data comparisons between EV studies. For instance, unpurified EV samples may be more accurately termed “extracellular biocolloidal particles” to take into account the mixture of colloidal particles produced by organisms, akin to the way humic substances are described.

Overall, this work provides a foundation upon which the transport capabilities of EVs can be built. α values were determined for three microbial EVs, and a model was developed from this data. Through a sensitivity analysis, the model is shown to be sensitive to both flow velocity and α, allowing for predictions of the transport distance in porous media with respect to these two parameters. In particular, the sensitivity of the model to changes in α can be utilized by researchers to identify what surface properties are needed to transport EVs to a target distance in the environment.

## Supporting Information Available

Experimental details and methods, including schematic of experimental setup (Figures S1–S4); additional EV characterizations (ζ-potential, size data, TEM images; Figures S5–S7); parameters and sample code for the model (Table S1 and Section S4); statistical results for ANOVA and Tukey’s post hoc test results (Tables S2–S22); and alternative visualizations of data (Figures S8 and S9) (PDF)
