# Supplementary material for: Characterizing the Transport and Surface Affinity of Extracellular Vesicles Isolated from Yeast and Bacteria in Well-Characterized Porous Media
Source: Environ Sci Technol. 2023 Aug 22;57(35):13182–92. doi: 10.1021/acs.est.3c03700 (PMC10483924; doi:10.1021/acs.est.3c03700)
Supplement: Supplementary file 1 — es3c03700_si_001.pdf [file es3c03700_si_001.pdf]

Supporting Information

Characterizing the Transport and Surface Affinity of Extracellular Vesicles Isolated from Yeast and Bacteria in Well-Characterized Porous Media

Author list: Nicholas M.K. Rogers*, Ethan Hicks, Christopher Kan, Ethan Martin, Lijia Gao, Clariss Limso, Christine Ogilvie Hendren, Meta Kuehn, Mark R. Wiesner*

Corresponding Authors:
Nicholas M.K. Rogers - Department of Mechanical Engineering, Porter School of Earth and Environmental Studies, Tel Aviv University, Tel Aviv, 69978, Israel; Email: nmkrogers093@tauex.tau.ac.il

Mark R. Wiesner - Center for the Environmental Implications of Nanotechnology, Department of Civil & Environmental Engineering, Duke University, Durham, North Carolina 27708, United States; Email: wiesner@duke.edu

Authors:
Ethan Hicks - Center for the Environmental Implications of Nanotechnology, Department of Civil & Environmental Engineering, Duke University, Durham, North Carolina 27708, United States

Christopher Kan – Department of Civil & Environmental Engineering, Duke University, Durham, North Carolina 27708, United States

Ethan Martin - Department of Civil & Environmental Engineering, Duke University, Durham, North Carolina 27708, United States

Lijia Gao - Department of Civil & Environmental Engineering, Duke University, Durham, North Carolina 27708, United States

Clariss Limso – Department of Biochemistry, Duke University Medical Center, Durham, North Carolina 27710, United States

Christine Ogilvie Hendren - Department of Geological and Environmental Sciences, Research Institute for Environment, Energy and Economics, Appalachian State University, Boone, NC, 28608, United States.

Meta Kuehn - Department of Biochemistry, Duke University Medical Center, Durham, North Carolina 27710, United States

Number of Pages : 14
Number of Figures : 9
Number of Tables : 22

S.1. Tracer Experiment Information

Tracer column experiments were performed as described in the Methods section in the associated publication. A schematic of this column setup is shown in Figure S1; sample data from this experiment is provided in Figure S2. Tracer tests using 10 mM KNO_3 through the column setup correspond well to column bypass tests (Figure S3). Small differences in the timing of the breakthrough curve from liftoff to reaching C_0 are likely due to dispersion of the tracer in the column and tubing compared to the column bypass. Figure S4 also indicates the relative absorbance of a background humic acid solution alone relative to the absorbance of the solution containing both EVs and humic acid.

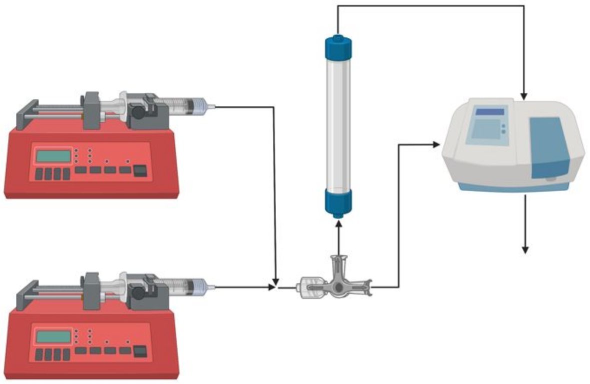

Figure S1: Schematic of column apparatus. Two syringe pumps are arranged to pump fluid into the column system: one contains the background electrolyte and one contains the EV sample. A valve is connected to the column inlet to allow for fluid flow directly to the in-line UV vis spectrophotometer for an initial concentration, or C0 reading. Created with Biorender.com.

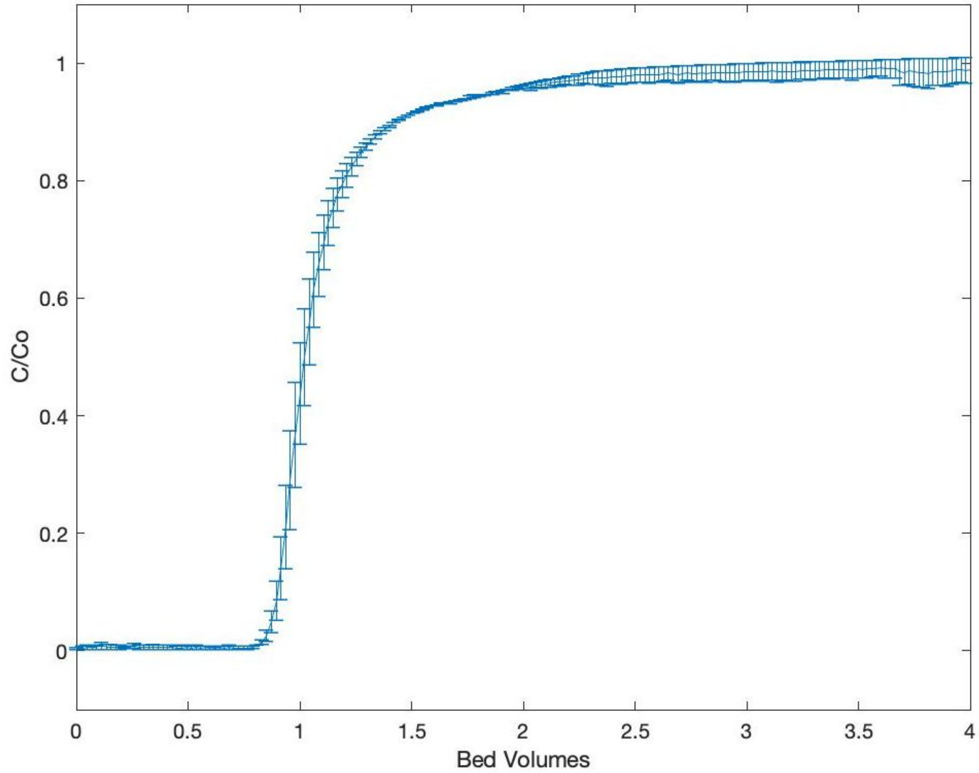

Figure S2: Sample Tracer Test Plot. Potassium nitrate is used as the tracer at 10 mM and at a 0.8 mL/min superficial velocity. Error bars are standard error for two replicates. Plateau is achieved at 2.25 bed volumes.

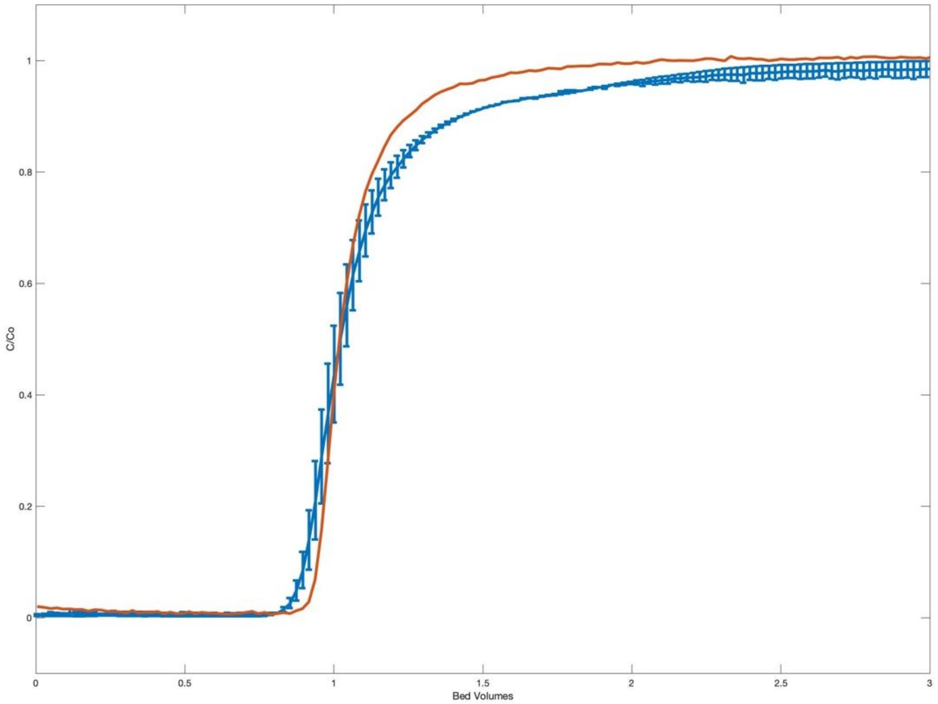

Figure S3: Comparison of tracer experiment through the column with the tracer bypassing the column. The tracer is 10 mM potassium nitrate. The orange line is the column bypass and the

blue line is the tracer passed through the column directly. Error bars for the data for the tracer through the column are standard error of two replicates. The small discrepancy between these two curves can be attributed to the dispersion within the column setup.

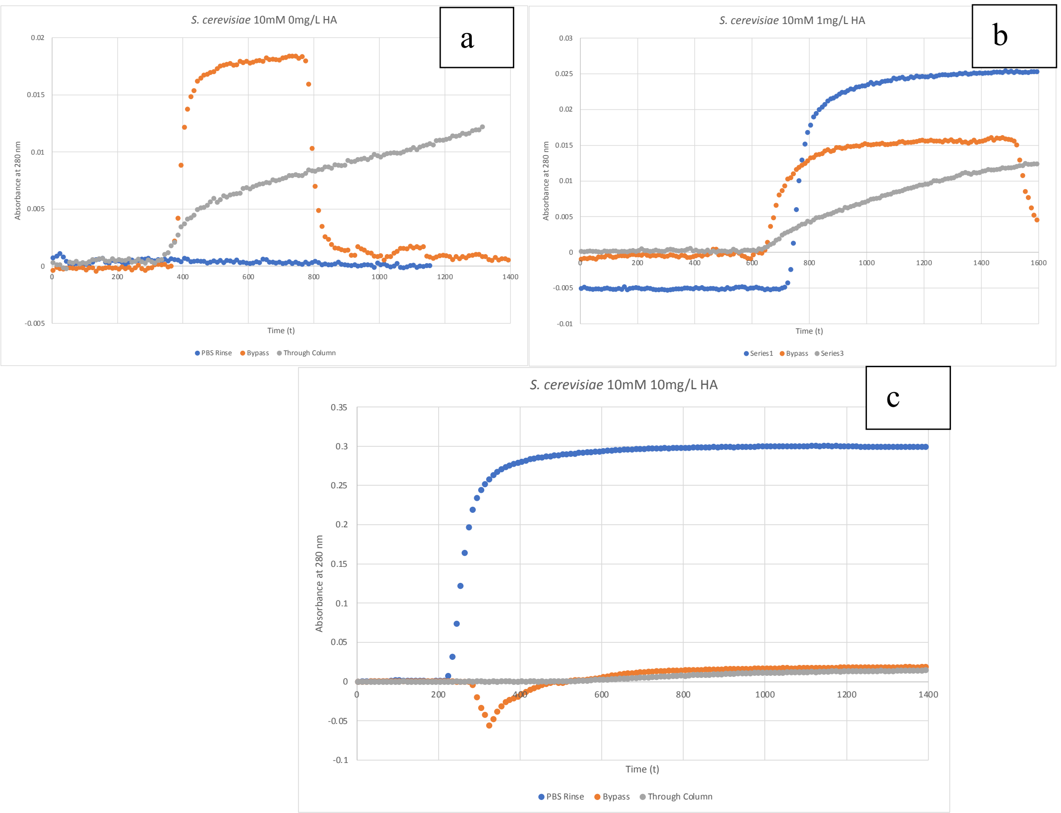

Figure S4: Raw absorbance data for electrolyte rinse of column, column bypass, and column test data in a) 0 mg/L HA, b) 1 mg/L HA, and c) 10 mg/L HA. All tests were performed at 10 mM ionic strength PBS with a superficial flow rate of 0.8 mL/min. As the humic acid concentration increases, the absorbance for the column test relative to the background becomes small. Note that each of these plots is presented to observe the relative difference between the absorbance of the background electrolyte relative to the sample in the column; time is irrelevant in these plots.

S.2. Additional EV Characterizations

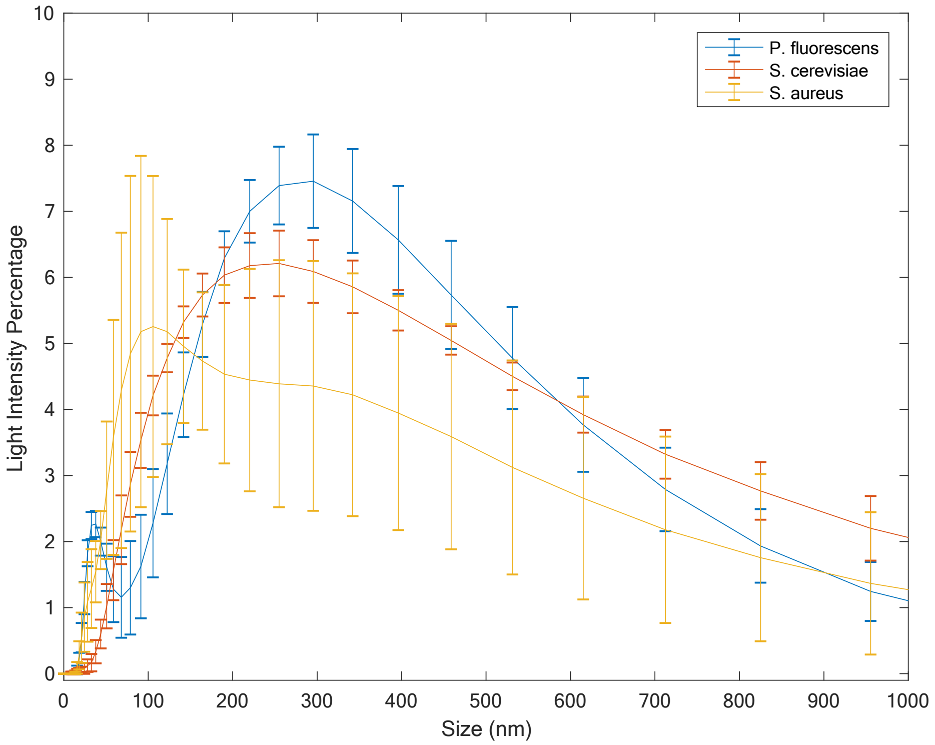

Figure S5. DLS data for EVs from all three organisms at pH 7 and 1 mM ionic strength. Error bars are standard error from three biological replicates. Light intensity was normalized to the light intensity for the most populous size bin for each respective sample.

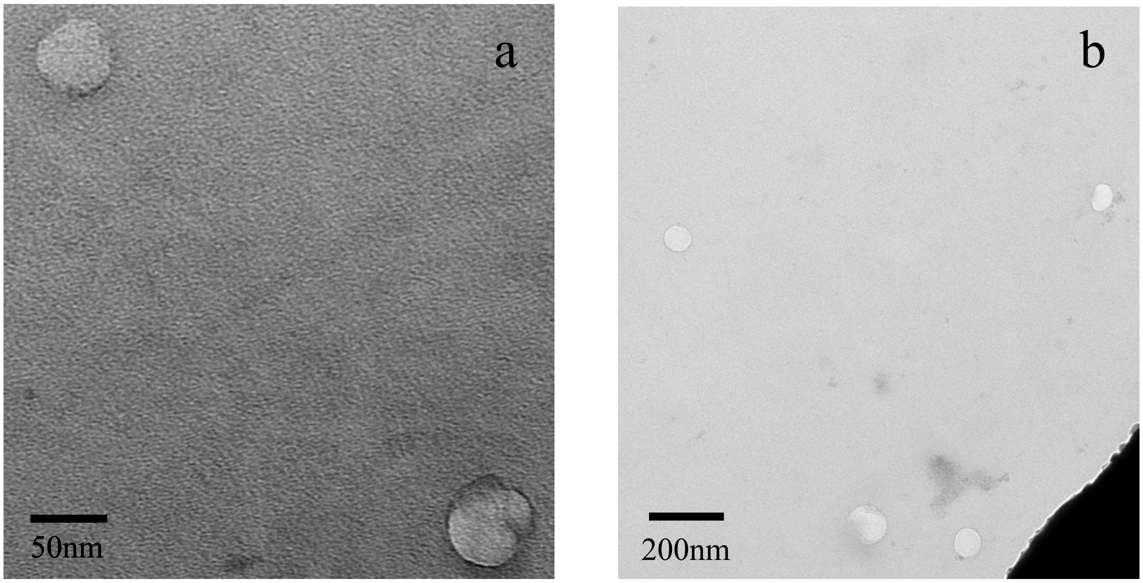

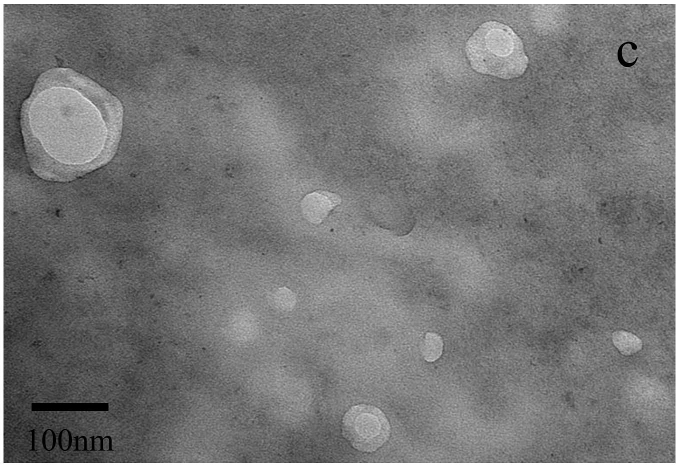

Figure S6. Negative-stained TEM images of EVs from a) *Pseudomonas fluorescens*, b) *Saccharomyces cerevisiae*, c) *Staphylococcus aureus*.

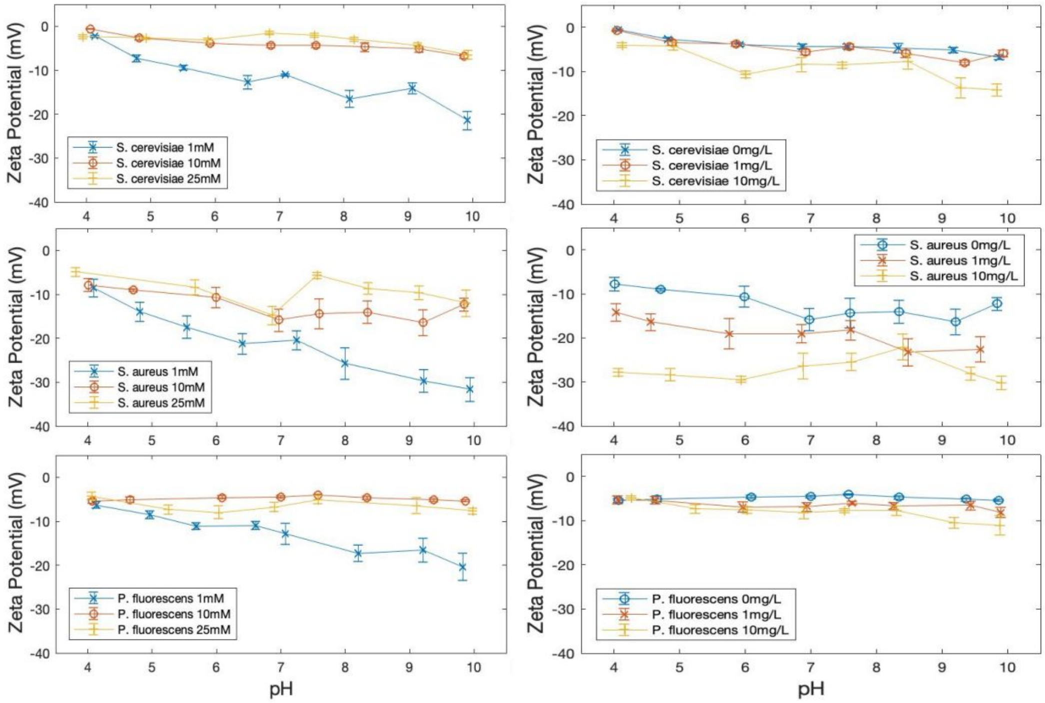

Figure S7: Zeta potential as a function of ionic strength and HA concentration for EVs from three organisms, error bars are standard error as described in the methods section. The listed ionic strength (in mM) is ionic strength of PBS; the listed mass concentration (in mg/L) is the concentration of HA. Data graphed in the left column were all with 0 mg/L HA; data graphed on the right were all measured using 10 mM PBS conditions.

S.3. Model Parameters

Table S1. Model parameters and values

Parameter	Value
Collector diameter	365 μm

EV diameter	123 nm
Temperature	25°C
Fluid density	1000 kg/m^3
EV particle density	1135 kg/m^3*
Viscosity	10^{-3} Pa*s
Porosity	0.32
Hamaker Constant	5 x 10^{-20} J
Flow velocity	1.69 x 10^{-4} m^3/s

*[59]

S.4. Model MATLAB Code

```
function [output] = VesicleTransportModel_v1(param)
% =========================================================================
% Vesicle Transport Model
% =========================================================================
% Nicholas M.K. Rogers and Ethan C. Hicks
% Duke University, Durham, N.C.
% Last Updated: February 2022
% =========================================================================
% Scope: To model the distribution of extracellular vescicles (EVs) through sediment.
% =========================================================================
% Instrucitons:
% =========================================================================
clear % to clear-out variable still in Workspace between runs

% -------------------------------------------------------------------------
% Input parameters (User action required):
% -------------------------------------------------------------------------
% velocities 3.8*10^-61.7*10^-4
% EV number con 1.03*10^21
% EV flow velocity 0.000169765
% dc to use for columns: 3.65*10^-4

param = [3.65*10^-4, 123*10^-9, 25, 1000, 1135, 10^-3, 1.7*10^-4, 0.32, 5*10^-20, 1.03*10^21, 0,
.0059];
dc          = param(1);    % Collector diameter (m)
dp          = param(2);    % Particle diameter (m)
T           = param(3);    % temp. (oC)
rho         = param(4);    % medium density (kg/m/m/m)
rho_p       = param(5);    % particle density (kg/m/m/m)
mu          = param(6);    % Dynamic or absolute viscosity (Pa*s)
v0          = param(7);    % Superficial velocity (m/s)
epsilon     = param(8);    % Porosity
A           = param(9);    % Hamaker constant (J)
n(1)        = param(10);   % Initial number concentration of EVs (#/m/m/m)
z(1)        = param(11);   % Initial distance (taken to be 0 as "at surface")
alpha       = param(12);   % Attachment efficiency (#/#)

% -------------------------------------------------------------------------
% Euler Integration (User action required):
% -------------------------------------------------------------------------
% Distance Parameter
startingz = -.15; % Starting position (m) (usually 0 as a reference point)
endingz = 0; % Ending position (m) (depth, with surface as reference point)
distance = endingz - startingz; % Total distance calculation (m)
% Euler Integration steps
M = 10000; % Number of steps
zstep = distance/M; % height/depth of each step

% -------------------------------------------------------------------------
% Model Selection (User action required):
% -------------------------------------------------------------------------
% Select model for calculation by subsituting any positive intiger in place
% of 0
Levich = 0;
Yao = 0;
Rajagopalan_Tien = 0;
Tufenkji_Elimelech = 1;
```

```
% -------------------------------------------------------------------------
% Constants:
% -------------------------------------------------------------------------
g = 9.81; % gravitation constant (m/s/s)
kB = 1.38 * 10^(-23); % Boltzmann constant (m*m*kg/s/s/K)
T_K = T+273.15;

% -------------------------------------------------------------------------
% Dimensionless Numbers
% -------------------------------------------------------------------------
N_R = dp/dc; % Aspect Ratio
N_Lo = 4*A/(9*pi*dp^2*v0); % London Attraction Number
N_G = dp^2*(rho_p-rho)*g/(18*mu*v0); % Gravitational Number
N_Pe = 3*mu*dp*v0*dc/(kB*T_K); % Peclet Number
gamma = (1-epsilon)^(1/3); % Filter Coefficient
A_s = 2*(1-gamma^5)/(2-3*gamma+3*(gamma^5)-2*(gamma^6)); %Porosity-dependent Number
N_vdw = A/(kB*T_K); % van der Waals Number
% N_A = A/(12*pi*mu*(dp/2)*(dp/2)*v0); % Attraction Number

% -------------------------------------------------------------------------
% Eta calculations
% -------------------------------------------------------------------------
% Levich (1962)
if Levich  > 0
Eta_B = 0.9*(kB*T_K/(mu*dp*dc*v0))^(2/3); % Brownian motion term
Eta_I = 0; % Shear motion term
Eta_G = 0; % Gravitational term
end
% Yao (1969)
if Yao > 0
Eta_B = 0; % Brownian motion term
Eta_I = (3/2)*((dp/2)/(dc/2))^2; % Shear motion term
Eta_G  = (rho_p-rho)*g*dp*dp/(18*mu*v0); % Gravitational term
end
% Rajagopalan and Tien (1976)
if Rajagopalan_Tien > 0
Eta_B = 4.04*A_s^(1/3)*N_Pe^(-2/3); % Brownian motion term
Eta_I = 0.72*A_s*(N_Lo^(1/8))*(N_R^(15/8)); % Shear motion term
Eta_G = (2.4*10^-3)*A_s*(N_G^1.2)*(N_R^-0.4); % Gravitaional term
end
% Tufenkji & Elimelech (2004)
if Tufenkji_Elimelech > 0
Eta_B = 2.4*(A_s^(1/3))*(N_R^-0.081)*(N_Pe^-0.715)*(N_vdw^0.052); % Brownian motion term
Eta_I = 0.55*A_s*(N_R^1.55)*(N_Pe^-0.125)*(N_vdw^0.125); % Shear motion term
Eta_G = 0.22*(N_R^-0.241)*(N_G^1.11)*(N_vdw^0.053); % Gravitational term
end
% Eta R calculation
Eta_T = Eta_B + Eta_I + Eta_G;
% etaT = 0.14 at .25 V
% etaT = 0.084 at .5 V
% etaT = 0.052 at  V

Eta_R = alpha*Eta_T;

% -------------------------------------------------------------------------
% Population Balance(s)
% -------------------------------------------------------------------------
for m = 1:M
% Linearized variables a and b
a = (-3/2)*((1-epsilon)*Eta_R/dc);
% Calculating change in population of free EVs as a function of depth, or
% distance (z).
z(m+1) = z(m)+zstep;
n(m+1) = n(m)+zstep*(a.*n(m));
end
[output] = (n);

% -------------------------------------------------------------------------
% Plots
% -------------------------------------------------------------------------
hold on
%yellow[0.9290 0.6940 0.1250];
%blue [0 0.4470 0.7410]
%red [0.6350 0.0780 0.1840]
plot(z,(n/n(1)),'Color','0.9290 0.6940 0.1250','LineWidth', 1.5)
```

```
set(gcf,'color','w')
set(gca,'fontsize',20)
set(gca,'TickDir','out')
ylabel("n/n0")
xlabel("Distance (m)")
xlim([0 .15])
ylim([0 1])
box on

w = n(10000)/n(1);
x=2;

%end
end
% ======================================================================
% END OF CODE
% ======================================================================
```

S.5 Statistical Testing Results

Table S2. ANOVA results for zeta potential data with respect to changes in ionic strength for *P. fluorescens* EVs.

Source	Sum of Squares	Degrees of Freedom	Mean Squares	F Statistic	P value
Media	338.8280963	2	169.4140481	8.52491415	0.00159514
Error	476.9475778	24	19.87281574		
Total	815.7756741	26			

Table S3. ANOVA results for zeta potential data with respect to changes in ionic strength for *S. aureus* EVs.

Source	Sum of Squares	Degrees of Freedom	Mean Squares	F Statistic	P value
Media	316.8134889	2	158.4067444	3.02326368	0.06745334
Error	1257.502578	24	52.39594074		
Total	1574.316067	26			

Table S4. ANOVA results for zeta potential data with respect to changes in ionic strength for *S. cerevisiae* EVs.

Source	Sum of Squares	Degrees of Freedom	Mean Squares	F Statistic	P value
Media	431.0414889	2	215.5207444	164.132723	1.00E-14
Error	31.51411711	24	1.313088213		
Total	462.555606	26			

Table S5. ANOVA results for zeta potential data with respect to changes in HA concentration for *P. fluorescens* EVs.

Source	Sum of Squares	Degrees of Freedom	Mean Squares	F Statistic	P value
Media	62.38895556	2	31.19447778	3.49606348	0.046507
Error	214.1458444	24	8.922743519		
Total	276.5348	26			

Table S6. ANOVA results for zeta potential data with respect to changes in HA concentration for *S. aureus* EVs.

Source	Sum of Squares	Degrees of Freedom	Mean Squares	F Statistic	P value
Media	533.1886741	2	266.594337	4.50870648	0.02175797
Error	1419.090844	24	59.12878519		
Total	1952.279519	26			

Table S7. ANOVA results for zeta potential data with respect to changes in HA concentration for *S. cerevisiae* EVs.

Source	Sum of Squares	Degrees of Freedom	Mean Squares	F Statistic	P value
Media	76.61025185	2	38.30512593	3.93635948	0.0332281
Error	233.5465111	24	9.73110463		
Total	310.156763	26			

Table S8. Tukey post hoc test results for zeta potential data with respect to ionic strength for *P. fluorescens* EVs.

Condition A	Condition B	Lower Limit of 95% Confidence Interval	Difference in Mean Values	Upper Limit of 95% Confidence Interval	P value
1 mM	10 mM	-13.613533	-8.3655556	-3.1175782	0.00154725
1 mM	25 mM	-11.426866	-6.1788889	-0.9309115	0.01888359
10 mM	25 mM	-3.0613107	2.18666667	7.43464404	0.55920325

Table S9. Tukey post hoc test results for zeta potential data with respect to ionic strength for *S. cerevisiae* EVs.

Condition A	Condition B	Lower Limit of 95% Confidence Interval	Difference in Mean Values	Upper Limit of 95% Confidence Interval	P value
1 mM	10 mM	-8.0367692	-6.6877778	-5.3387863	1.92E-11
1 mM	25 mM	-10.881214	-9.5322222	-8.1832308	6.13E-15
10 mM	25 mM	-4.1934359	-2.8444444	-1.495453	6.12E-05

Table S10. Tukey post hoc test results for zeta potential data with respect to HA concentration for *P. fluorescens* EVs.

Condition A	Condition B	Lower Limit of 95% Confidence Interval	Difference in Mean Values	Upper Limit of 95% Confidence Interval	P value
0 mg/L	1 mg/L	-1.1909512	2.32555556	5.84206231	0.24424606
0 mg/L	10 mg/L	0.16460436	3.68111111	7.19761786	0.03896525
1 mg/L	10 mg/L	-2.1609512	1.35555556	4.87206231	0.60691565

Table S11. Tukey post hoc test results for zeta potential data with respect to HA concentration for *S. aureus* EVs.

Condition A	Condition B	Lower Limit of 95% Confidence Interval	Difference in Mean Values	Upper Limit of 95% Confidence Interval	P value
0 mg/L	1 mg/L	-5.8534693	3.19888889	12.2512471	0.65629011
0 mg/L	10 mg/L	1.55764184	10.61	19.6623582	0.01946303
1 mg/L	10 mg/L	-1.6412471	7.41111111	16.4634693	0.1232763

Table S12. Tukey post hoc test results for zeta potential data with respect to HA concentration for *S. cerevisiae* EVs.

Condition A	Condition B	Lower Limit of 95% Confidence Interval	Difference in Mean Values	Upper Limit of 95% Confidence Interval	P value
1 mM	10 mM	-2.4190104	1.25333333	4.92567704	0.67480131
1 mM	25 mM	0.3587674	4.03111111	7.70345482	0.02952148
10 mM	25 mM	-0.8945659	2.77777778	6.45012149	0.16359744

Table S13. ANOVA results for C/C_0 values with respect to changes in ionic strength for *P. fluorescens* EVs.

Source	Sum of Squares	Degrees of Freedom	Mean Squares	F Statistic	P value
Media	0.002051284	2	0.001025642	1.14272259	0.42762149
Error	0.002692627	3	0.000897542		
Total	0.00474391	5			

Table S14. ANOVA results for C/C_0 values with respect to changes in ionic strength for *S. aureus* EVs.

Source	Sum of Squares	Degrees of Freedom	Mean Squares	F Statistic	P value
Media	0.086363314	2	0.043181657	1.7318042	0.31620543
Error	0.074803474	3	0.024934491		
Total	0.161166789	5			

Table S15. ANOVA results for C/C_0 values with respect to changes in ionic strength for *S. cerevisiae* EVs.

Source	Sum of Squares	Degrees of Freedom	Mean Squares	F Statistic	P value
Media	0.178491056	2	0.089245528	127.754	0.00125018
Error	0.00209572	3	0.000698573		
Total	0.180586776	5			

Table S16. ANOVA results for C/C_0 values with respect to changes in HA concentration for *P. fluorescens* EVs.

Source	Sum of Squares	Degrees of Freedom	Mean Squares	F Statistic	P value
Media	0.028689082	2	0.014344541	14.1766928	0.02959751
Error	0.003035519	3	0.00101184		
Total	0.031724601	5			

Table S17. ANOVA results for C/C_0 values with respect to changes in HA concentration for *S. aureus* EVs.

Source	Sum of Squares	Degrees of Freedom	Mean Squares	F Statistic	P value
Media	0.018484833	2	0.009242416	0.32887831	0.7427791
Error	0.084308536	3	0.028102845		
Total	0.102793369	5			

Table S18. ANOVA results for C/C_0 values with respect to changes in HA concentration for *S. cerevisiae* EVs.

Source	Sum of Squares	Degrees of Freedom	Mean Squares	F Statistic	P value
Media	0.060485367	2	0.030242683	10.3155198	0.04523323
Error	0.008795296	3	0.002931765		
Total	0.069280663	5			

Table S19. Tukey post hoc test results for C/C_0 data with respect to ionic strength for *S. cerevisiae* EVs.

Condition A	Condition B	Lower Limit of 95% Confidence Interval	Difference in Mean Values	Upper Limit of 95% Confidence Interval	P value
1 mM	10 mM	0.25036597	0.36081187	0.47125778	0.00174129
1 mM	25 mM	0.26030022	0.37074612	0.48119203	0.00160633
10 mM	25 mM	-0.1005117	0.00993425	0.12038015	0.92690308

Table S20. Tukey post hoc test results for C/C_0 data with respect to HA concentration for *P. fluorescens* EVs.

Condition A	Condition B	Lower Limit of 95% Confidence Interval	Difference in Mean Values	Upper Limit of 95% Confidence Interval	P value
0 mg/L	1 mg/L	-0.127211026	0.005711778	0.13863458	0.98247758
0 mg/L	10 mg/L	0.016535756	0.14945856	0.28238136	0.03672718
1 mg/L	10 mg/L	0.010823977	0.143746781	0.27666959	0.04073027

Table S21. Tukey post hoc test results for C/C_0 data with respect to HA concentration for *S. cerevisiae* EVs.

Condition A	Condition B	Lower Limit of 95% Confidence Interval	Difference in Mean Values	Upper Limit of 95% Confidence Interval	P value
0 mg/L	1 mg/L	-0.353738972	-0.127478736	0.0987815	0.19007296
0 mg/L	10 mg/L	-0.472142142	-0.245881906	-0.0196217	0.04020793
1 mg/L	10 mg/L	-0.344663407	-0.11840317	0.10785707	0.21959161

Table S22. ANOVA results for C/C_0 values with respect to changes D'Arcy velocity.

Source	Sum of Squares	Degrees of Freedom	Mean Squares	F Statistic	P value
D'arcy Velocity	0.000350676	2	0.000175338	0.08688693	0.91900509
Error	0.006054001	3	0.002018		
Total	0.006404677	5			

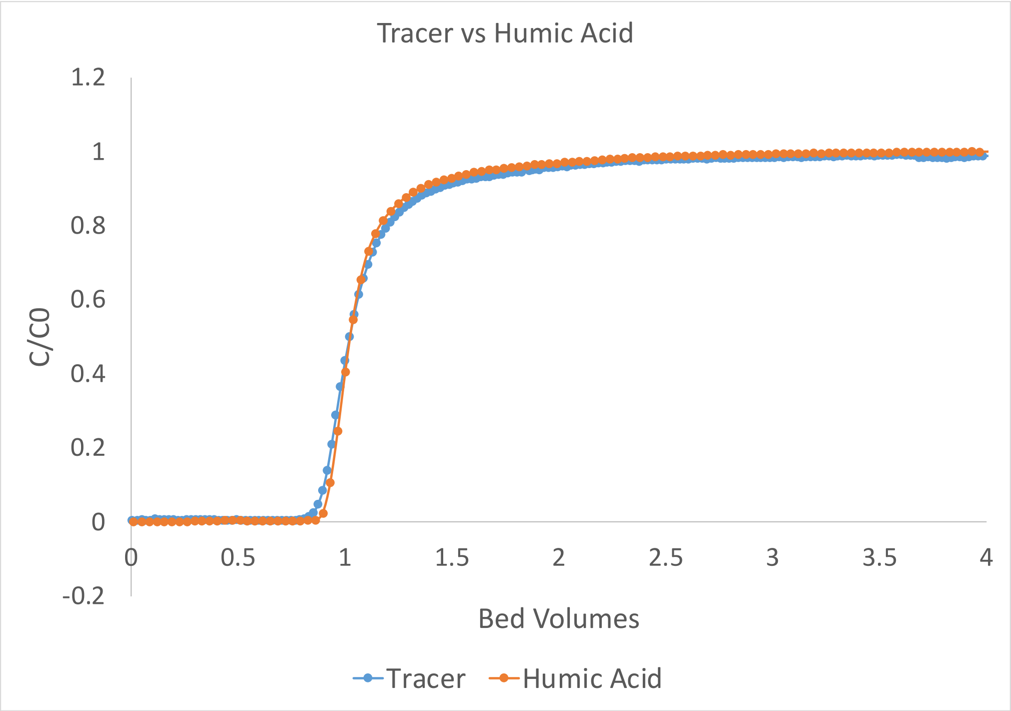

Figure S8. Comparison of the tracer experiment with and without humic acid. The tracer is 10 mM potassium nitrate. The humic acid is not being retained in the column.

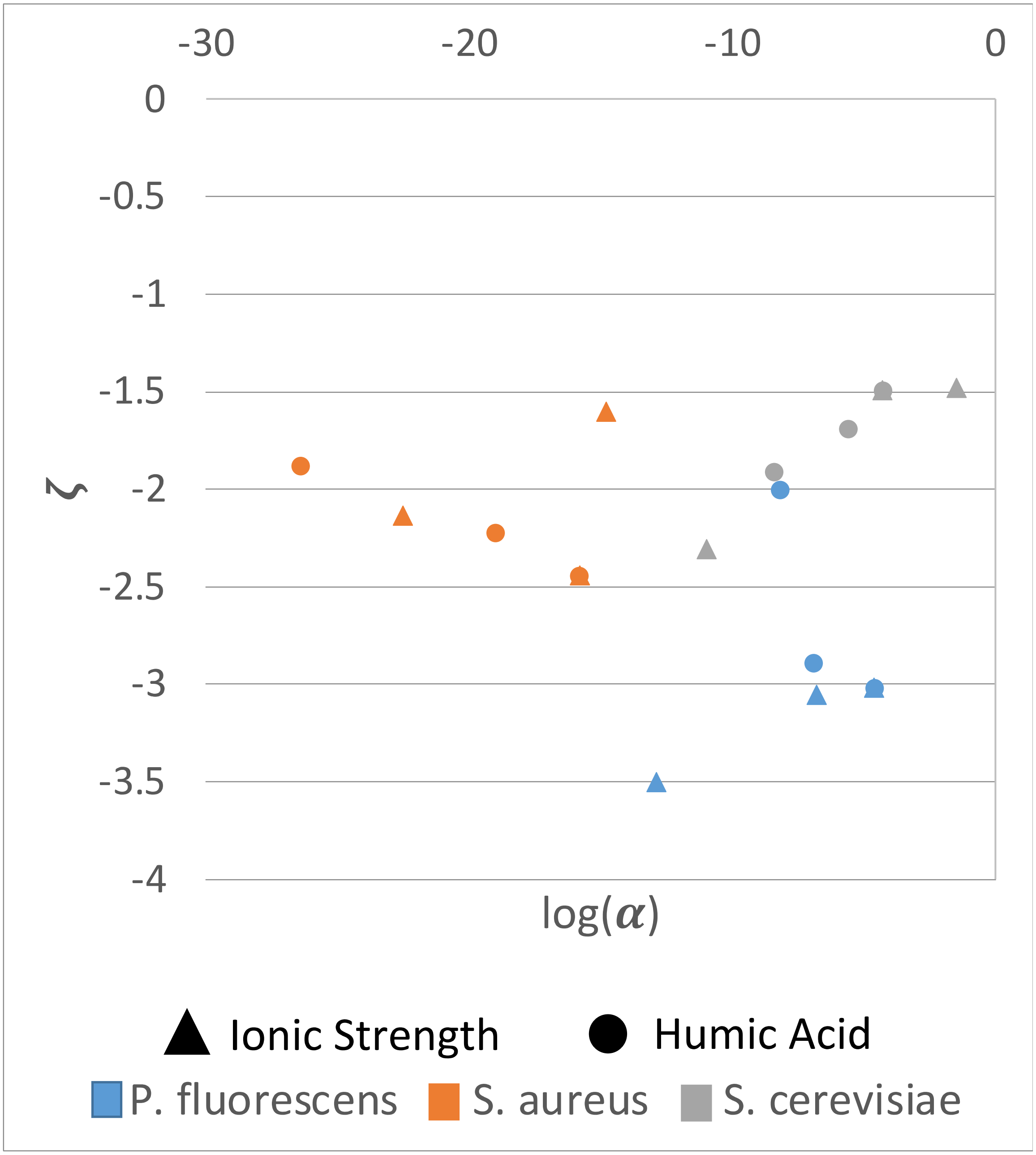

Figure S9. Log of the attachment efficiency (α) of each of the three types of EVs as a function of zeta potential (ζ). Triangles are for the change in these parameters as a function of ionic strength; circles are for the change in these parameters as a function of humic acid concentration.
